# Current News

**Published:** 1900-12

**Authors:** 


### Current News.

### BOARD OF EXAMINERS OF PENNSYLVANIA.

THE Board of Dental Examiners of Pennsylvania will conduct examinations simultaneously in Philadelphia and Pittsburg, December 17 to 20, 1900.

Application for examination must be made to Hon. James W. Latta, Secretary of the Dental Council, Harrisburg, Pa.

G. W. KLUMP,

*Secretary.*

WILLIAMSPORT, PA.

### THE TROUBLE IN THE NEW JERSEY STATE BOARD.

[VOLUMINOUS matter has been received relating to the controversy between certain members of the State Examining Board of New Jersey and Dr. Charles A. Meeker, another member. It would require too much space to reproduce the entire subject, and, therefore, an abstract of the charges against Dr. Meeker is appended, together with the closing of Dr. Meeker in his full answer to the charges sent to the governor of the State of New Jersey.—ED.]

CHARGES.

1. Dr. Meeker is charged with being unfit for the position he occupied.

2. "In almost every case (Dr. Meeker) gave a candidate a passing vote."

3. "The board became convinced that Dr. Meeker was not voting according to the papers written by the applicants under examination. . . . Proof was not secured till at the July, 1899, meeting of the board."

4. "Dr. Meeker has seriously hampered the work of the board, not only by his failure to attend meetings, but in his neglect in preparing questions for the examination."

The foregoing, in full was sent to the governor, signed by Geo. E. Adams, D.D.S., President; F. C. Barlow, D.D.S., E. M. Beesley, D.D.S., E. Carlton Brown, D.D.S., Secretary.

The governor thereupon sent the following notice to Dr. Charles A. Meeker:

"STATE OF NEW JERSEY, EXECUTIVE DEPARTMENT,
"October 5, 1900.

"DEAR SIR,—Charges of incompetency and inefficiency as a member of the State Board of Registration and Examination in Dentistry have been laid before me. A copy thereof is hereto annexed.

"Before disposing of the same it is but fair that you should be given an opportunity to reply thereto, and I have accordingly fixed the hour of ten A.M., on the 13th day of October, 1900, at my office in Elizabeth, as the time and place when I will proceed to consider the same, and hear you if you so desire.

"Yours truly,
"FOSTER M. VOORHEES.

"CHARLES A. MEEKER, D.D.S."

In reply to this request Dr. Meeker sent a very full answer to the charges, an abstract of which is herewith appended.

"The first specific charge is that in almost every case I gave a passing vote. In reply to this, I assert that my vote as to the merits of the papers presented by candidates has in all cases represented my honest conviction as to the merits of these papers.

"The charge that I have hampered the work of the board, not only by my failure to attend meetings, but by my neglect in preparing questions for the examination is absolutely false.

"The charge that I have invariably given candidates a passing vote would mean that I have always given candidates as many as twenty votes. The true facts may be ascertained by an examination of the records."

Dr. Meeker then closes as follows:

"I desire to say, generally, that for a number of years I served in this Association with the gentlemen who now prefer charges against me with perfect accord and harmony, and during all that time our Commission was a member of the National Association, and it was conceded that we were exerting a wide influence through that body for the elevation of the standards of professional education throughout the country. Dr. G. Carleton Brown was one of the most arduous workers in the National Association, and the rules of that body, which he afterwards insisted should operate to make us resign our membership, were formulated largely under his influence and direction. Very suddenly and without apparent reason my associates have exhibited personal hostility to me, and they now allege 'incompetence.' It is to be noted that this incompetence is a matter of recent discovery, and the specifications contained in the charges are pitifully weak and frivolous. The majority of the members of the board have attempted to force me to resign my membership in it, and were bitterly chagrined at my re-election in 1899. I have been at a loss to understand why this change of feeling towards me, and but one explanation has occurred to me. Some years ago, when serving on the auditing committee, I expressed surprise that no vouchers were presented for the sums expended. I did this purely as a matter of the proper conduct of the business of the board, but it was resented as a personal affront, and I was not again appointed on the auditing committee, and since then I have without difficulty discovered the hostility towards me which has been exhibited by my associates.

"I have been for several years the Secretary and Treasurer of the National Association, and have been for twenty-five years the Secretary of the State Dental Society, and have devoted a large part of my time and have incurred considerable expense in advancing the interest of the profession in this State and throughout the country. I have been re-elected to office by the National Association, and also by the State society, and if I am incompetent for membership of the State Commission, such incompetency must have existed during all these years, and must be proved by something more than loosely worded charges and malicious criticism.

"As an example of the petty annoyances to which I have been subjected by my associates, I will state that on May 3, 1900, I was notified to attend a meeting of the State board for Saturday, May 5, at eight P.M. It was known then to my associates in the board that a banquet tendered by the president to the officers of our local society had been set for that date, and that I had in a measure assumed the management of the arrangements. This notice was sent to me by registered mail, the only instance of the registration of a notice of meeting. It happened that the banquet of the president was postponed to Saturday, May 12, and this becoming known I was promptly notified that the meeting of the Commission had been postponed to May 12, and this explains my absence from one meeting. It also illustrates the purpose of the conduct of my associates in their treatment of me.

"Again, a meeting of the State board was called for July 28, the day on which I was to sail for Europe as a delegate to the International Congress of Dentists, having been appointed by the State society and also by the National Association as their representative. After my departure for Europe, a change was made in the subjects of the various examiners. An examination of these changes shows that two of the examiners kept their old subjects; a third taking Dr. Beesley's, while mine was taken from me and given to Dr. Osmun. This is an unusual proceeding, the custom in our board and other examining boards being to continue the subjects of each examiner so long as he remains an examiner, with a view to securing the highest service from the board.

"Much more might be added in explanation of the animus of these charges which your Excellency is now forced to consider, and I am grateful to you for the opportunity of presenting my answer thereto; but I respectfully submit that in no single particular have I failed to perform my official duty as a member of the examining board, and that it must be clear that personal hostility and not devotion to the interests of a great profession lies at the bottom of the action of my associates. They probably believe that even though these charges shall be dismissed by your Excellency, they will nevertheless have succeeded in doing me a professional injury, and it is not unlikely that they will be satisfied with this as the solitary result of their action in presenting these charges.

"All of which is respectfully submitted.

"CHAS. A. MEEKER."

Dismissed by the governor and exonerated.

### CENTRAL DENTAL ASSOCIATION OF NORTHERN NEW JERSEY—RESOLUTIONS OF CONFIDENCE IN DR. MEEKER.

Whereas, Charges of incompetency and inefficiency have been lodged with the governor of this State against a member of the Board of Registration and Examination in Dentistry; and

Whereas, Such member of the Board of Registration and Examination in Dentistry is also a member of this society in good standing and of good repute; and

Whereas, Such member having practised dentistry in this State for more than thirty years, and during that time been one of the most progressive and active members of the profession,—having held all the offices of honor that could be bestowed by both the State and Central societies,—who before the operation of the present dental law was for years the State prosecutor of violators of the dental law, the duties of which office he discharged with marked ability and discretion; who has been honored with office by the National Association of Dental Examiners, and who has been regarded for many years by the profession in this State and practically every State in the United States, and in many parts of South America and Europe, as a professional man of ability and attainments, one whose professional course was worthy of emulation by the younger members of the profession; one who by his fidelity to trusts imposed upon him has done more to elevate and advance the interests and good name, as well as fame, of the New Jersey State Dental Society and the Central Dental Association of Northern New Jersey than any other member of either of the societies, and one who has never before been charged with dereliction or neglect in the discharge of official duties, it seems right and proper that we, the members of the Central Dental Association of Northern New Jersey, in regular meeting assembled on this fifteenth day of October, 1900, in the city of Newark, N. J., should give public expression of our feelings and sentiments concerning the charges that have been made against our fellow member and treasurer, Dr. Charles A. Meeker, of Newark; therefore be it

*Resolved,* That we, the members of the Central Dental Association of Northern New Jersey, express to Dr. Charles A. Meeker, a member of this society, our confidence in his professional ability, his fidelity to duty, and his untiring devotion to the cause of dental education and advancement; and be it

*Resolved,* That a copy of these resolutions be forwarded to Hon. Foster M. Voorhees, governor of this State, and also spread upon the minutes of this society.

---

### AMERICAN DENTAL SOCIETY OF EUROPE.

At the twenty-seventh annual meeting of the American Dental Society of Europe, held in Paris upon August 7, 1900, the following-named officers were elected for the ensuing year: President, Dr. W. Mitchell, London; Vice-President, Dr. S. S. Macfarlane, Frankfort-on-Main; Honorary Treasurer, Dr. L. J. Mitchell, London; Honorary Secretary, Dr. W. E. Royce, Tunbridge Wells.

*Executive Committee.*—Dr. L. J. Mitchell, Dr. E. Lawley-York, Dr. J. H. Spaulding, Dr. L. C. Bryan, Dr. W. S. Davenport, Dr. W. L. Croll. Ex-officio members, Dr. W. Mitchell, Chairman; Dr. W. E. Royce, Honorary Secretary.

*Membership Committee.*—Dr. C. J. Monk, Dr. G. Cunningham, Dr. W. L. Croll. Ex-officio members, Dr. S. S. Macfarlane, Chairman; Dr. W. E. Royce, Honorary Secretary.

Waldo E. Royce,
*Honorary Secretary.*

---

### INSTITUTE OF DENTAL PEDAGOGICS.

The eighth annual meeting of the Institute of Dental Pedagogics will convene on Thursday, December 27, 1900, at ten o'clock A.M., at the Maxwell House, Nashville, Tenn., and will hold sessions on December 27, 28, and 29.

Henry W. Morgan,
David M. Cattell,
Walter E. Willmott,
*Executive Committee.*

